# Vobi One: a data processing software package for functional optical imaging

**DOI:** 10.3389/fnins.2014.00002

**Published:** 2014-01-24

**Authors:** Sylvain Takerkart, Philippe Katz, Flavien Garcia, Sébastien Roux, Alexandre Reynaud, Frédéric Chavane

**Affiliations:** ^1^Institut de Neurosciences de la Timone UMR 7289, CNRS - Aix Marseille UniversitéMarseille, France; ^2^LabISEN, Vision Department, Institut Supérieur de lElectronique et du NumériqueBrest, France; ^3^McGill Vision Research, Department of Ophtalmology, McGill UniversityMontréal, QC, Canada

**Keywords:** python, neuroscience, optical imaging, linear model, signal processing

## Abstract

Optical imaging is the only technique that allows to record the activity of a neuronal population at the mesoscopic scale. A large region of the cortex (10–20 mm diameter) is directly imaged with a CCD camera while the animal performs a behavioral task, producing spatio-temporal data with an unprecedented combination of spatial and temporal resolutions (respectively, tens of micrometers and milliseconds). However, researchers who have developed and used this technique have relied on heterogeneous software and methods to analyze their data. In this paper, we introduce *Vobi One*, a software package entirely dedicated to the processing of functional optical imaging data. It has been designed to facilitate the processing of data and the comparison of different analysis methods. Moreover, it should help bring good analysis practices to the community because it relies on a database and a standard format for data handling and it provides tools that allow producing reproducible research. *Vobi One* is an extension of the BrainVISA software platform, entirely written with the Python programming language, open source and freely available for download at https://trac.int.univ-amu.fr/vobi_one.

## 1. The optical imaging technique

Functional optical imaging is a large family of brain imaging techniques that consist in measuring changes of an optical property that is directly or indirectly related to changes of neural activity. As shown in several reviews (Hillman, 2007; Kim and Jun, 2013), these techniques span a very wide range of spatial scales, from microscopic to macroscopic, and can be applied on *in vitro* preparation, in anesthesized and awake animals *in vivo* and even non-invasely in humans. Here, we focus on a sub-group of this family, namely *in vivo* mesoscopic functional optical imaging using intrinsic or fluorescent signal. The imaging data produced with these particular techniques share common properties that make them approachable by similar data processing strategies. First, the *in vivo* functional approach generally sets the experimental paradigm to trial-based protocols designed to study sensory-evoked responses. Second these neural responses are recorded at the mesoscopic scale, i.e., within one brain area with a micrometric spatial resolution and a temporal resolution in the order of milliseconds. We now very briefly review the most relevant techniques that belong to this sub-group.

A first technique consists in examining optical properties that are intrinsic to the studied tissue, as for instance the scattering, the autofluorescence or the absorption. Among these, the absorption is the main intrinsic optical property that is used *in vivo*, mainly to image the topographic organization of the cortex (Grinvald et al., 2001). Since the absorption spectra of oxy- and deoxy-hemoglobins are different, it is indeed possible to map local changes in blood oxygenation level that are indirectly related to changes in neural activity (Iadecola, 2004).

In contrast, extrinsic optical imaging is based on the use of an external fluorescent dye that is added to the neural tissue. In mesoscopic imaging, one can use voltage-sensitive dyes that linearly reflect changes in membrane potential into modulations of the fluorescence level (Shoham et al., 1999). These dyes therefore act as probes of the membrane potential integrated over the total membrane surface that has been stained. Until now, voltage-sensitive dye imaging (VSDI) is the only technique that allows to directly record the neural activity over a large neural population (of up to 2 × 2 cm) with an unprecedented combination of spatial resolution (tens of micrometers) and temporal resolution (milliseconds), in both anesthetized and awake preparations (Grinvald and Hildesheim, 2004).

### 1.1. Signal and noise in mesoscopic functional optical imaging

With these techniques, the fluctuations in optical properties that are induced by neural activity are very small. They are riding over a general baseline level of absorption or fluorescence that is, in general, about a thousand times larger. Furthermore, there exist many sources of noise that corrupt the signal in a comparable, or even larger, range than the functionally-evoked fluctuations. These noise sources are of different origin: instrumental (noise in the photo-detector itself, lack of stability of the light source), physiological (heart beat, breathing, vasomotion), or environmental (illumination homogeneity, dye bleaching, external sources of vibrations); see (Grinvald et al., 2001) for a review. It is therefore crucial to develop strategies that improve both the experimental protocols themselves and the data processing methods. The first option, improving the experimental protocols, has already proven successfull [see (Shoham et al., 1999; Kalatsky and Stryker, 2003; Yang et al., 2007; Vanni et al., 2010; Onat et al., 2011; Omer et al., 2013)]. Below, we quickly review the various data processing strategies that have been developed over the last decades. It is to be noted that none has yet led to a consensus, and as a consequence, the results reported in different published studies may not be fully comparable.

### 1.2. Data processing techniques

#### 1.2.1. Standard method

The standard method used to process data from functional optical imaging experiment consists in two successive normalization steps (Shoham et al., 1999). The first one, known as *frames 0* division, normalizes to the baseline level by dividing each image of the stack by the first frames (recorded before stimulus onset). It relies on the assumption that changes of absorption or fluorescence are linearly dependent on the baseline level. The second one compares trials with and without stimulation (the latter being referred to as *blank trials*). In order to remove the effects of noise sources that are synchronized with data acquisition (in general, dye bleaching, heart-beat and respiration-triggered acquisition), image stacks collected during stimulated trials are subtracted by those acquired during blank trials on a frame-by-frame basis. These operations generate fractional signals, generally denoted $\frac{\Delta F}{F}$ for fluorescence. Optionally, a third step can be added to remove residual slow drifts induced by dye bleaching with a linear detrending of the timeseries (Chen et al., 2008; Meirovithz et al., 2009).

This framework yields satisfactory results in studies for which the noise level is low, i.e., in stable anesthetized preparations for which the signal is averaged over many trials. However, when working in behaving animals and/or at the single-trial level, serious limitations persist. The first one is that many sources of noises are not fully removed, either in the spatial domain (for instance vascular patterns) or in time (physiological oscillations for instance). The second one is that the initial division by the mean of the *frames 0* is an inaccurate normalization method that biases the estimation of the variability over time (Takagaki et al., 2008; Reynaud et al., 2011). Below, we describe the two main families of methods that have recently been applied on optical imaging data to overcome the aforementioned limitations; both rely on the principle that the recorded signal is a linear combination of a limited number of sources.

#### 1.2.2. Source separation techniques

Blind source separation techniques aim at estimating the different sources contained in a signal based on their statistical properties. Among these, the main families of algorithms are Principal Components Analysis [PCA; (Cannestra et al., 1996; Gabbay, 2000; Sornborger et al., 2003)], Independent Component Analysis [ICA; (Brown et al., 2001; Maeda et al., 2001; Inagaki et al., 2003; Reidl et al., 2007)] and Extended Spatial Decorrelation [ESD; (Stetter, 2000; Schiessl et al., 2008)]. These techniques convincingly proved to be accurate in denoising optical spatial maps (for instance to remove blood vessels in activation maps). But they all require to perform an a posteriori classification of the components into signal sources (i.e., containing the neural response) or noise sources, which is often not trivial. Moreover, nothing guarantees the accuracy of the source separation: some components may include both signal and noise.

To overcome these problems, new decomposition methods with Indicator Function (IF) have been proposed: images are projected onto orthogonal subspaces containing signal and noise sources, determined by comparing stimulated and blank trials. IF has been applied on intrinsic imaging recorded in anesthetized animals (Everson et al., 1997; Gabbay, 2000; Yokoo, 2001). Alternatively, other methods use a priori knowledge about the temporal structure of the response, for instance by constraining the experimental protocol using periodic stimulation. Under such conditions, the response can be extracted by modeling the oscillations by a periodic function (Le and Hu, 1996; Mitra and Pesaran, 1999) or by using methods such as the temporally structured component analysis (TSCA) algorithm (Blumenfeld, 2010; Omer et al., 2013).

Most of these methods rely on the assumption of signal separability between space and time. However, VSDI data, with its combination of very high spatial and temporal resolution, often includes components which violate this hypothesis: indeed the temporal shape of the neural response can vary over space, and in a symmetric manner, the localization of noise-induced patterns can evolve in time. Some of the aforementioned methods propose different sets of regression matrices to allow some variability (Blumenfeld, 2010). However, these methods assume that the shape of the response is stable and are not designed to extract a continuous range of response patterns, which is a strong limitation for single-trial analysis.

#### 1.2.3. Multiple linear regression

A solution which is by essence designed to be applied on a trial-by-trial basis is the multiple linear regression technique. Initially developed for PET and functional MRI, it has recently been applied to optical imaging of intrinsic signal (Rector, 2001; Zheng et al., 2001; Bathellier et al., 2007), blood flow (Mayhew et al., 1998), calcium fluorescence (Stetter, 2001) or synapto-phluorin fluorescence (Bathellier et al., 2007). This technique requires the a priori identification of the physical sources of signals in order to define regressors that are included in the model. Such method guarantees a better separation of noise and signal components and discount for any bias in components selection.

We recently introduced a method that allows constructing such a linear model, with the aim of extracting the activity dynamics in the extrinsic VSDI signal. The reader is invited to read (Reynaud et al., 2011) for the full details of the method, which goes beyond the scope of this paper, but we will now summarize our framework (which is also described in Section 3.1.2). It starts with a stepwise characterization of all the noise components contributing to the raw VSD signal collected in the primary visual cortex of behaving macaques. Then, we incorporated a set of basis functions that optimally describe a large number of plausible response shapes, as determined by sensible ranges for several shape-controlling parameters. With this model we have been able to precisely estimate a large range of dynamic shapes for the neural response evoked by different visual stimuli. The output of our framework was systematically compared with the standard techniques described above. We demonstrated that our model provides better denoising results than these traditional methods, over the spatial, temporal and trials dimensions (Reynaud et al., 2011). Moreover, we showed in (Reynaud et al., 2012) that this method can be successfull at the single trial level on behaving monkey data and in a large variety of stimulation conditions (with differences in contrast, duration and location). We believe that it can be easily generalized to less noisy preparations like anesthetized animals and will allow new findings from optical imaging experiments.

## 2. *Vobi one*: a software perspective

The vast heterogeneousity of methods dedicated to the processing of functional optical imaging datasets and the lack of a common platform to make these methods available and comparable led us to design such a software tool. In details, we had three main objectives when we designed *Vobi One*:

- To facilitate the data processing itself, for instance by offering standardized workflows and by allowing for easy comparisons between different methods.
- To help bringing good analysis practices to the users, i.e., by using a standard data format, a database to store and retrieve files.
- To provide a package that could be used both through a graphical user interface (GUI) and by writing scripts in order to fit the needs of all kinds of users, independently of their computing skills.

Furthermore, a software suite that would fill all these specifications will also facilitate providing reproducible research, following most of the principles described in Sandve et al. (2013), and ease interactions between different research groups working on functional optical imaging data.

The choice was made to develop *Vobi One* as a toolbox of the BrainVISA software platform. In what follows, we detail how this choice allows reaching the aforementioned objectives.

### 2.1. The BrainVISA software environment

BrainVISA^1^ is an open source software platform dedicated to the analysis of neuroimaging data (Geffroy et al., 2011). It comes with a comprehensive library of image processing functions and tools, that are accessible either by calling executables (compiled for each operating system) or through wrapper libraries callable from different programming languages such as C, C++, and Python. Moreover, the BrainVISA environment offers several characteristics that directly meet some of our specifications.

First, its data management strategy relies on the use of a database to index data which is stored in the filesystem. The ontology of the database is customizable (as described below in 2.2) and BrainVISA insures the equivalence between the database organization and the architecture of the filesystem (name and organization of directories, subdirectories and files). The current version of BrainVISA uses SQLite to organize and manage its database, allowing for a standalone use (i.e., without any server) and direct access from Python.

Second, BrainVISA supports several file formats that are commonly used in the neuroimaging community, including the recently developed Nifti1.0 file format. The Nifti format has been designed to provide a unique data format that would be supported by all neuroimaging data analysis packages, which allows for easy exchanges between different software platforms. Although Nifti was thought to store magnetic resonance imaging data, it is perfectly adapted to store data acquired in functional optical imaging experiments. We therefore decided to use the Nifti1.0 format to store all the data that needs to be manipulated in *Vobi One*.

**Figure 1:**
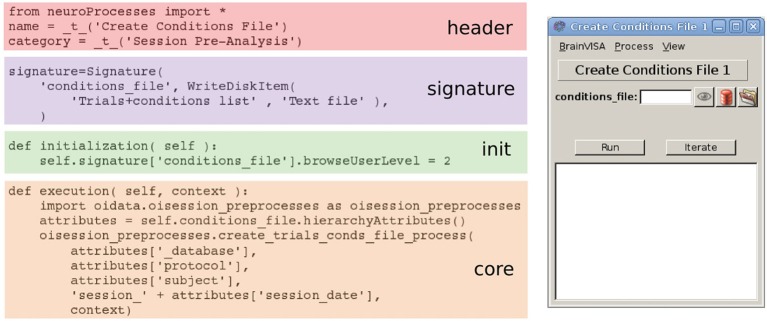
**Illustration of the use the BrainVISA API with one of the *processes* defined in *Vobi One***. The source code (from which comments have been removed for space purposes) follows a simple formatting with four sections. It produces the GUI displayed on the right. Note that the core section only contains a call to a single function, which is a generic coding principle we adopted in *Vobi One*.

Third, BrainVISA comes with an application programming interface (API) that makes it possible to easily develop GUIs, as illustrated on Figure 1. Indeed, constructing a GUI with the BrainVISA API consists in writing a Python script that mainly describes the inputs and outputs. Following this format, a view is automatically generated for the user to fill in the input parameters; the outputs are automatically named according to the filesystem architecture that matches the ontology of the database. A panel is also created to allow the user to follow the execution of the process.

On top of these three features which directly meet some of our needs, *Vobi One* will directly benefit from two extra capabilities offered by BrainVISA. First, it is possible to define specific viewers for each type of data handled by BrainVISA; in particular, BrainVISA comes with a companion software, Anatomist, which is dedicated to visualizing imaging data. Second, BrainVISA provides tools that facilitates the processing of large amounts of data: (1) the *iterate* function allows to repeat the same analysis on different datasets, i.e., to set up a loop from the GUI without having to write a program; (2) another companion package of BrainVISA, Soma-workflow^2^, is a simple interface to parallel computing ressources such as multi-core machines and compute clusters.

### 2.2. *Vobi one*: a BrainVISA toolbox

A BrainVISA toolbox is a set of Python^3^ programs divided into three components, each living in a subdirectory: *types, hierarchies*, and *processes*. The toolbox defines the GUI for each analysis component, as well as the customized database ontology used to store and retrieve data files.

The different files that need to be manipulated by the toolbox are defined in the *types* subdirectory. Precisely, three different object types are defined here: *FileType, Format*, and *HierarchyDirectoryType*. A large list of these three types of objects is provided by the BrainVISA environment. The *Format* entries defined in a toolbox like ours allow defining new file formats which are not recognized by BrainVISA, with their associated file extension. In the case of *Vobi One*, we defined several proprietary file formats used by the optical imaging companies that are included in the acquisition set-ups in functional optical imaging experiments. The *HierarchyDirectoryType* define directory types as used in the toolbox. Finally, the *FileType* entries define the categories of files used and created by the toolbox; for instance the type “OI GLM Denoised” was created to identify the files containing the result of a denoising using a general linear model (GLM). All these entries are defined in an object-oriented fashion and can therefore inherit properties from their father *FileType*, in particular from the ones natively defined in BrainVISA.

The *hierarchies* files define the way the data is stored on disk in correspondence with the SQLite BrainVISA database. Two types of entries are therefore described in the *hierarchies*: (1) the architecture and naming conventions of directories and sub-directories, which start from the root BrainVISA database directory, and which match the *HierarchyDirectoryType* defined in *types*; and (2) the naming conventions for all the files stored in these directories, which match the *FileType* entries defined in *types*.

Finally, the heart of the toolbox is a set of *processes*. Each process corresponds to an elementary operation. As illustrated on Figure 1, it is defined in one Python file that includes four parts: (1) some header information; (2) the so called *signature* that defines the shape of the GUI for this process, with the list of inputs and outputs, and their corresponding *FileType* entries; (3) an initialization function that sets up default parameters and dependencies between the values of the input and output fields; and (4) the core of the process that defines the operations performed when the process is launched from the user interface. BrainVISA also offers the capability to create *pipelines* that combine a set of elementary *processes*; this feature makes it easy to design complex workflows that consists in executing a sequence of several operations.

**Figure 2:**
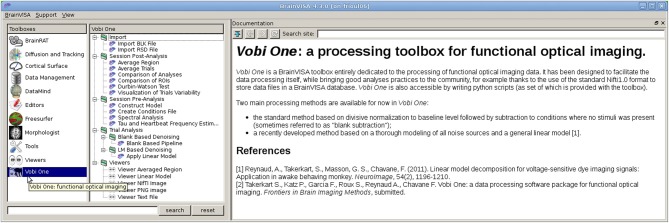
**The main *Vobi One* interface, as part of BrainVISA**. *Vobi One* is listed and selected in the list of toolboxes in the **left** panel. The **middle** panels shows all the available *processes*. The **right** panel displays the associated documentation.

Once these three types of Python files are fully defined, the toolbox appears directly integrated in the main BrainVISA user interface, as illustrated in Figure 2 with our *Vobi One* toolbox.

### 2.3. *Vobi one*: functionalities

As described in Section 2.2, each elementary operation is callable by a *process* of the toolbox. The *processes* are grouped into several categories, which follow the structure of the suggested analysis workflows which will be described in detail in Section 3. The main *Vobi One* panel (see Figure 2) is embedded inside the BrainVISA user interface and allows picturing the organization of the toolbox. In what follows, we examine the content of each category of *processes*.

#### 2.3.1. Importing routines

In functional optical imaging experiments, the acquired data is composed of timeseries of two-dimensional images (2D+t data). Each experimental trial is stored in a single file, in a format, often proprietary, provided by the CCD camera vendors. For now, *Vobi One* includes importing routines for two of these formats (.blk files for Optical Imaging Ltd., and .rsd files for SciMedia USA Ltd.), which are the ones used in the setups at our institute and that are also widely used by the community. All importing routines have the same functions: (1) they allow to convert data from the input format into the standard Nifti 1.0 format; (2) they populate the BrainVISA database so that all the following processes can efficientely retrieve the data; and (3) the Nifti files stored in the database all follow the same naming convention, regardless of the input format; this name includes all major informations about the experimental protocol (the date of acquisition, the experience number within that day, the trial number, and a number that encodes the characteristics of the stimulus for this trial). Finally, in order to reduce the data volume, it is possible to directly zip the Nifti files, and also to bin the data temporally or spatially if the resolution of the raw data is unecessarily high, which will also reduce the processing time. Note that if one needs to import data files that are not supported at the moment, the necessary information is provided in the documentation for developers who would like to add the necessary importing routines.

#### 2.3.2. Session preprocessing routines

This set of processes are preparatory steps common for the analysis of all trials of a single session.

***2.3.2.1. Creation of the “Condition file.”***. Once your data is imported, this process creates a text file, called “Condition file” that summarizes the information about all imported trials. It is mostly redundant with the keys of the database ontology itself, but allows for interfacing with external software that cannot directly access the database. Moreover, it is used by several subsequent processes.

***2.3.2.2. Construction of the linear model***. In order to analyze single trials with the linear model, one need to define the set of regressors that will be included in the design matrix of the multiple linear regression (see Section 3.1.2). Three *processes* are available to facilitate this construction. The first one (called “Tau and Heartbeat frequency estimation”) allows estimating the rate of the exponential decay in the timeseries (dye bleaching), as well as the strongest sinusoıdal component present in the signal, which is often caused by the heart beat. The second *process* (called “Spectral Analysis”) identifies peaks in the the Fourier spectrum of the timeseries that are induced by noise-related oscillatory components present in the raw signal. These first two processes helps to define the characteristics of the noise components that are included in the model. The third *process* (called “Construct model”) actually creates the full design matrix containing all the regressors from the noise sources characteristics estimated with the previous two *processes* and the expected shape of the neural response itself.

#### 2.3.3. Trial analysis processes

The *processes* available in this category are the ones actually performing the analyses. Since they work at the single-trial level, they are designed to be used with the *iterate* capability in order to repeat the same analysis on all the trials of a given session. For now, two main classes of methods are available in *Vobi One* among the three described in Section 1.2: the blank-based standard method and the linear model-based method introduced in (Reynaud et al., 2011). As explained in 1.2.1 the standard blank-based method is a sequence of normalization steps, either substractive or divisive. In *Vobi One*, the blank-based method (implemented through a *pipeline*) starts by the *frames 0* division, and is followed by the *blank subtraction* and a *linear detrending*. Other individual processes are also available if one want to change the normalization method for each of these steps. For the linear model, one *process* is available to launch the estimation of the model parameters at each pixel; this *process* also computes the denoised version of the signal, i.e., the original signal from which the contributions of all the noise components have been removed.

#### 2.3.4. Session postprocessing routines

Several post-processing functions are available at the session level. Most of them consist in averaging results obtained at the single trial level, across all or a subset of trials of the selected session. For instance, *processes* are available to compare the results within one region of interest across different experimental conditions, or across several regions of interest, or to compare different models and/or methods. Another *process* allows to visualize the variability of the neural response estimated across trials. An additional goal of all these processes is to produce graphs that are directly usable in publications, as illustrated in Figure 5. Finally, an additional *process* performs a statistical test (the Durbin-Watson test) that checks whether the residuals of the linear model are white, which is an *a posteriori* indicator of the validity of the model itself (Reynaud et al., 2011).

#### 2.3.5. Visualization

The visualization tools available in *Vobi One* are defined as BrainVISA *viewers* (see Section 2.2). They are directly accessible in the user interface of any of the *processes* described above as soon as a viewer is available for the *file type* associated with an input or output parameter. Otherwise, they are accessible as standalone *processes* in the Viewers category. All image types are directly associated with Anatomist, the companion software of BrainVISA dedicated to image data visualization. Moreover, several viewers have been developed for the graphics produced in the session-level postprocessing routines (some of them are shown on Figure 5).

### 2.4. Scriptability and the oidata python module

As described above, the *Vobi One* toolbox sets up the computing environment around the processing workflows. One still needs to write the code that actually performs the different operations. In order to do this, several options are available: (1) include the code directly in the Python files that define the *processes* of the toolbox; (2) create executables that will be called from BrainVISA; (3) design a dedicated software library. The first option is definetely to be avoided because, for example, it would be impossible to share functions across different *processes*, resulting in duplicating code. The second option is the one used in most of the toolboxes that are included in the main BrainVISA platform. The developer can then choose its favorite programming language. The main advantage is that the executables can be used outside of BrainVISA. But an important drawback is that one has to go through a compilation process which can be cumbersome when attempting to offer a truely cross platform tool. We therefore opted for the third option, i.e., to develop a software library that would be linked to the toolbox code. Since the toolbox itself is written in Python, the easiest choice was to develop a dedicated Python module. We named it *oidata*, which stands for optical imaging data. We will now describe the advantages and drawbacks of this choice.

The main negative aspect when implementing some data analysis procedure in Python is the computational efficiency, or potential lack thereof compared with executables compiled from C or C++ source. But since most computations in *Vobi One* consists in linear algebra operations performed on matrices, its computational efficiency will be guaranteed by using *numpy*^4^ methods on *numpy* arrays (Van Der Walt et al., 2011).

On the other hand, developing a Python module offers several advantages: (1) no compilation is necessary to install *Vobi One*; the installation process simply consists in unpacking the pure Python module and the toolbox Python files into the main BrainVISA installation directory; (2) it seamlessly becomes possible to fully operate *Vobi One* by writing scripts in Python and directly calling the methods offered in this module.

Moreover, we imposed an extra constraint when designing the *oidata* Python module, by forcing that the core of each *process* in the *Vobi One* toolbox (as defined in 2.2) consists in calling a single function of the *oidata* module, as illustrated on Figure 1). Therefore, there is a perfect correspondance between using the GUIs and writing scripts. We hope that this can ease the task of users who start using *Vobi One* with the GUIs and want to start writing scripts because the structure of the scripts they would have to write would intuitively correspond to what they are used to do with the GUI. The example scripts provided with the toolbox illustrate this characteristic.

### 2.5. Interoperability

Another major feature of *Vobi One* is its possibility to interact with other softwares. It is indeed crucial that one can easily perform some operations with *Vobi One*, and some others, which for example are not available in *Vobi One* yet, by using other software tools. This interoperability capability is insured by the fact that all image stacks in *Vobi One* are stored in the Nifti format. Indeed, standard input/output libraries are available to manipulate Nifti files from many languages including C, Matlab and Python, and the Nifti format is also natively supported by numerous other software suites dedicated to neuro-imaging. Various analysis results provided by *Vobi One*, such as denoised timeseries averaged across all trials of a sessions for a given list of experimental conditions, can therefore be easily imported in other software environments.

## 3. *Vobi one*: workflows and example

In this section, we describe the main processing methods available in *Vobi One*. Overall, the analysis concept followed in our toolbox consists in setting up a processing framework that is common to all trials acquired during a given experimental session, and then to perform post-processing operations by gathering results from a large number of trials. We also provide illustrations of their application on a real dataset.

### 3.1. Workflows

**Figure 3:**
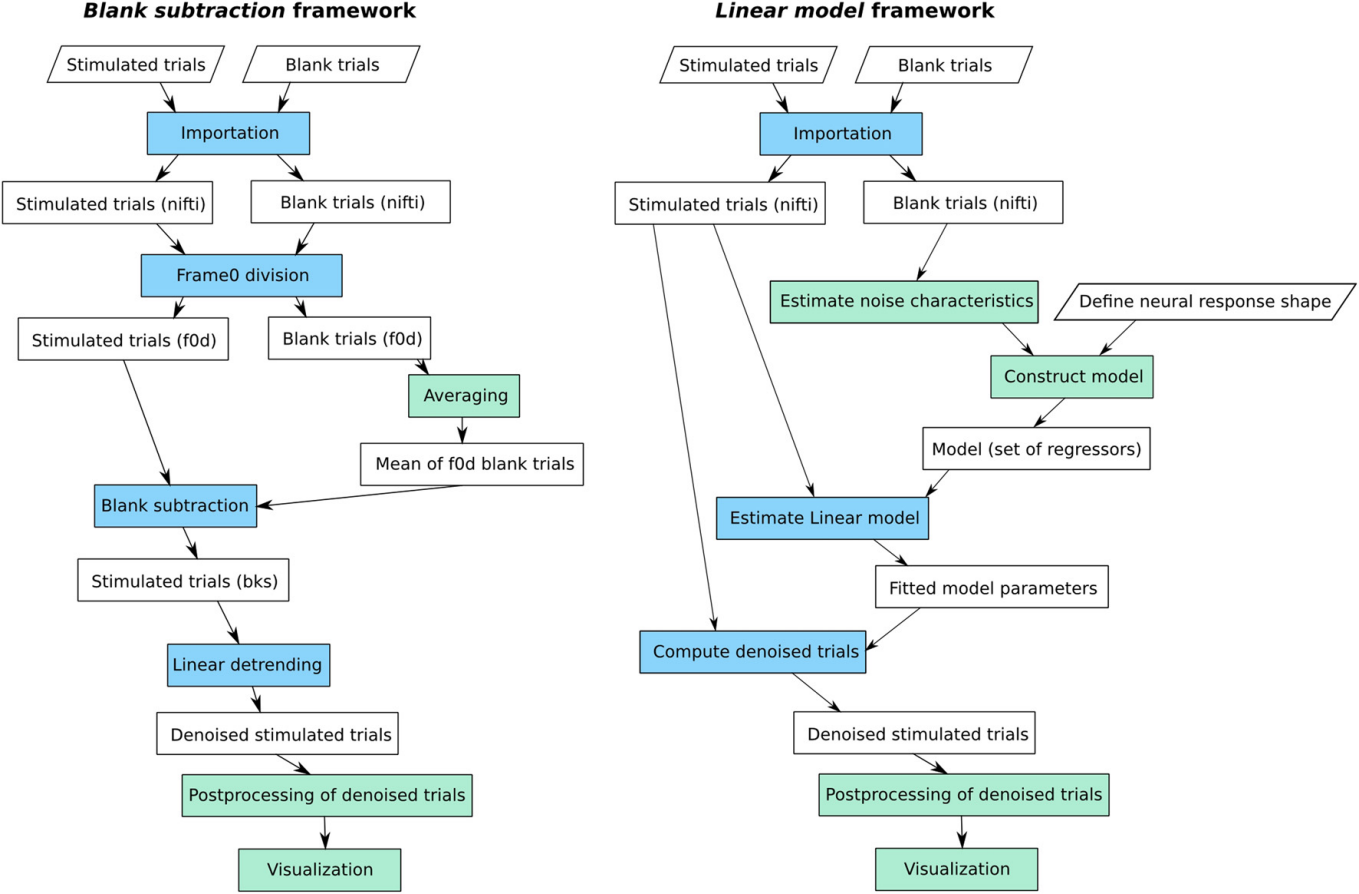
**The two main workflows available in *Vobi One*: the standard *blank subtraction* method (on the left), and the *linear model* from (Reynaud et al., 2011) (on the right)**. In green: session-level processes; in blue: trial-level processes, repeated on each individual trial.

Figure 3 shows the workflows available in *Vobi One* to implement the standard *blank subtraction* and our *linear model* frameworks. It depicts the chain of elementary operations which are described hereafter; all these operations correspond to a *process* available in *Vobi One* (as already described in Section 2.3).

#### 3.1.1. Blank subtraction

As described in 1.2.1, our implementation of the standard *blank subtraction* method consists in performing a sequence of normalization steps that use blank trials to provide a reference level and eliminate noise sources. For each individual trial, the first step consists in dividing the full image stack by the baseline level; this baseline level is estimated by averaging the first frames of the trial, recorded before the onset of the stimulus (the so called *frames 0*). Then, the average of all blank trials available for this session is computed. The result of this averaging process is subtracted from each stimulated trial. Finally, a linear detrending is performed to ensure that the estimated neural response comes back to baseline level at the end of each trial, producing the denoised trials on which $\frac{\Delta F}{F}$ is directly measured.

#### 3.1.2. Linear model

The linear model framework available in *Vobi One* consists in performing a multiple linear regression *y* = *X*β + ϵ, independently at each pixel and for each trial, in order to estimate the different components that add up to produce the raw timeseries *y*. The model contained in the design matrix *X* must therefore include a predictor (or regressor) for each potential component. Our previous work (Reynaud et al., 2011) described how to build such a set of predictors. In short, our model takes the following form:

$$y=X_{0}\beta_{0}+X_{n}\beta_{n}+X_{s}\beta_{s}+\epsilon$$

**Figure 4:**
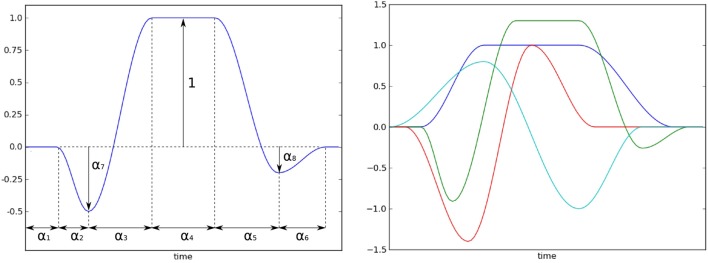
**Left: the model of the neural response shape with its parameter**. **Right**: several shape prototypes that can be obtained with the model. The user should define this prototype, and then chooses intervals for the parameters that allows for variations of the shape around the prototype.

- *X*_0_ is a constant regressor, which means that β_0_ is an estimate of the baseline level.
- *X*_*n*_ contains the different sources of noise. For VSDI signals, we showed that the noise components are of two nature: oscillatory components that can be modeled as Fourier series, and the dye bleaching that is modeled by a decaying exponential. In the workflow available in *Vobi One*, we propose to estimate some of the characteristics of these noise components from the data, and in particular the frequencies of the noise-induced oscillations that are estimated by computing the power spectrum of the timeseries of blank trials.
- *X*_*s*_ describes the shape of the neural response itself. In order to build a model that can accomodate for shape variations, we start from a generic model *r* that can be tuned to obtain very diverse chapes (as illustrated on Figure 4):
  $$r(t)=\{\begin{array}{ll} 0 & \text{if }t\leq \alpha_{1},\\ \frac{\alpha_{7}}{2}\text{​}\left(\cos\left(\pi \frac{t-\alpha_{1}}{\alpha_{2}}\right)-1\right) & \text{if }0<t-\alpha_{1}\leq \alpha_{2},\\ \frac{1-\alpha_{7}}{2}-\frac{1+\alpha_{7}}{2}\cos\text{​}\left(\pi \frac{t-\alpha_{1}-\alpha_{2}}{\alpha_{3}}\right) & \text{if }0<t-\alpha_{1}+\alpha_{2}\leq \alpha_{3},\\ 1 & \text{if }0<t-\alpha_{1}+\alpha_{2}+\alpha_{3}\leq \alpha_{4},\\ \frac{1-\alpha_{8}}{2}+\frac{1+\alpha_{8}}{2}\cos\text{​}\left(\pi \frac{t-\alpha_{1}-\alpha_{2}-\alpha_{3}-\alpha_{4}}{\alpha_{5}}\right) & \begin{array}{l} \text{if }0<t-\alpha_{1}+\alpha_{2}+\alpha_{3}\\ \;+\alpha_{4}\leq \alpha_{5}, \end{array}\\ -\frac{\alpha_{8}}{2}\left(1+\cos\text{​}\left(\pi \frac{t-\alpha_{1}-\alpha_{2}-\alpha_{3}-\alpha_{4}-\alpha_{5}}{\alpha_{6}}\right)\right) & \begin{array}{l} \text{if }0<t-\alpha_{1}+\alpha_{2}+\alpha_{3}\\ \;+\alpha_{4}+\alpha_{5}\leq \alpha_{6}, \end{array}\\ 0 & \begin{array}{l} \text{if }t>\alpha_{1}+\alpha_{2}+\alpha_{3}\\ \;+\alpha_{4}+\alpha_{5}+\alpha_{6} \end{array} \end{array}$$
  The user specifies the minimum and maximum values for each parameter α_*i*_ to define the range of plausible shapes for the current experiment. *Vobi One* then generates a very large set of shapes by browsing all combinations of values within these intervals, performs a principal component analysis on this set, and retains the *L* first components to define *X*_*s*_ (*L* being defined by the user).
- ϵ is the residual timeseries.

Once the model *X* is fully defined, it is fitted to the data (i.e., the β_*i*_ parameters are estimated to minimize ϵ) and the denoised timeseries are estimated by removing the noise components from the original timeseries and normalizing by the baseline level:

$$\frac{\Delta F}{F}=\frac{1}{\beta_{0}}\left(y-X_{n}\beta_{n}\right)$$

### 3.2. Example

We here illustrate some results obtained with *Vobi One* on a real dataset described below. Note that this dataset is distributed with the toolbox.

#### 3.2.1. Dataset

The data provided with *Vobi One* consists of a subset of the data that was used to validate the linear model in (Reynaud et al., 2011). The reader is referred to this article for a full description of the experimental protocol, but we here briefly describe the characteristics of this dataset that are relevant for the present paper. The data was recorded on an awake behaving monkey (*Macaca mulatta*) in a VSDI experiment. The CCD camera produced images with 512 × 512 resolution at an acquisition frequency of 110 Hz. For each trial, the beginning of the recording was triggered on the heartbeat of the animal as soon as it achieved correct fixation on the red dot located in the middle of the screen. A visual stimulus consisting of a drifting grating was then displayed on the screen. Six different contrast levels were used for the grating, changing from trial to trial. The dataset includes 10 trials per contrast level, as well as 20 trials where no stimulus was presented (blank trials), totaling for 80 trials. Each trial is provided as an image stack in a single file.

#### 3.2.2. Application of Vobi one

**Figure 5:**
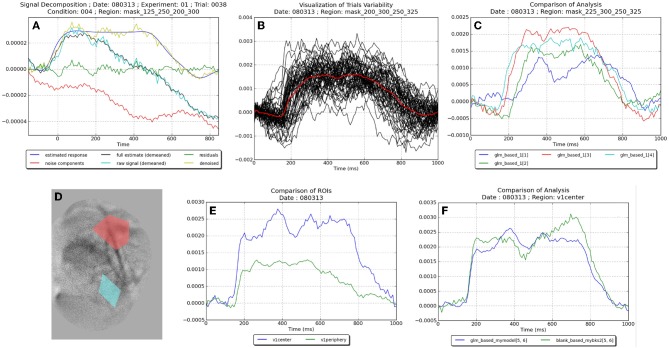
**Examples of figures produced by the different viewers and postprocessing routines on the VSDI dataset provided with *Vobi One***. **(A)** Illustration of the components (noise, neural response etc.) estimated by our linear model. **(B)** Denoised timecourses estimated with our linear model on all trials of a session. **(C)** Denoised timecourses obtained with four different contrasts of the visual stimuli. **(D)** Spatial map of the hearbeat contribution, with two regions of interest. **(E)** Denoised timecourses averaged in these two regions. **(F)** Comparaison of the denoised timecourses obtained with two analysis methods.

The tutorials contained in *Vobi One*'s user manual describe how to run the two aforementioned workflows, as well as several postprocessing operations. Figure 5 presents some of the results that can directly be produced by *Vobi One*:

- Figure 5A presents the details of the components estimated by our linear model on a single trial, averaged within a region of interest; it shows the raw timeseries, the estimated noise components and neural reponse, the timeseries of residuals and the denoised signal.
- Figure 5B shows the denoised timecourses obtained on all trials of a given session with the linear model.
- Figure 5C presents the denoised timecourses (estimated with our linear model) for four of the six experimental conditions (i.e., four contrast values).
- Figure 5D is a spatial map of the heartbeat contribution (estimated with our linear model), with two regions defined by the user; the regions can be manually drawn in Anatomist, or functionally defined by using the results provided by *Vobi One*.
- Figure 5E displays the denoised timecourses of the two regions shown in Figure 5D.
- Finally, Figure 5F compares the two analysis methods (the blank subtraction and the linear model).

## 4. Discussion

Our decision to develop a software package dedicated to the analysis of functional optical imaging data was initially motivated by the need to provide support for users at our institute. In practice, this meant replacing a set of Matlab scripts that had been developed and customized by several users, by a unique software package for which we would offer centralized support and that could also serve as the central piece of inhouse collaborative research. We recently decided to make *Vobi One* available to the community because it now contains enough functionalities to be an asset to the few groups using this modality within the neuroscience community, as we described earlier in this paper. In particular, we find it important that our central methodological contribution, the construction of a linear model to analyze single experimental trials, can be easily used by researchers in our community, which is now the case thanks to *Vobi One*.

### 4.1. Potential for other optical imaging techniques

Although the development of *Vobi One* has been motivated to answer the needs of the community of users of *in vivo* mesoscopic functional optical imaging techniques, we here span the other members of the large family of optical imaging techniques and ask whether our toolbox could be useful in each case.

At one extreme of this large family are techniques that directly measure neuronal activity at microscopic scales, such as two-photon imaging of calcium fluorescent signals (Grewe and Helmchen, 2009). At the other extreme, we find techniques operating at macroscopic scales such as functional near-infrared spectroscopy (Bunce et al., 2006; Ferrari and Quaresima, 2012) or event-related optical signals (Gratton and Fabiani, 2001). These two types of techniques rely on very different data acquisition and processing strategies and we do not believe that it would be sound to try to extend *Vobi One* in these directions.

Other types of techniques, like those which are extensively used *in vitro* such as optogenetic using fluorescent proteins (Knöpfel et al., 2010; Jin et al., 2012), light scattering (Cohen et al., 1972; Stepnoski et al., 1991) and intrinsic fluorescence (Shuttleworth, 2010), could easily benefit from the current software package with appropriate adaptations [see also the application of a GLM to *in vivo* data acquired using genetically expressed fluorescent protein in (Bathellier et al., 2007)]. Similarly, *Vobi One* could be thought of being generalized to recently developed techniques in intrinsic optical imaging of brain tissues such as laser speckle based techniques (Hillman, 2007) or direct observations of the blood flow (Deneux et al., 2011, 2012).

### 4.2. On-going developments in *Vobi one*

The development of *Vobi One* is still on-going, because all the features that are needed by the researchers are not yet available. At present, the way our local users process their data is by using *Vobi One* for all available operations and complementing it with their own code and tools for all unsupported tasks, thus demonstrating the inter-operability capability offered by the toolbox. We here want to briefly describe the main features that are currently under development.

First, although several processes are available to visualize timeseries, and compare them across regions, experimental conditions or processing methods, the visualization capability of the associated spatial maps still rely on a proficient use of Anatomist, the companion software of BrainVISA that is dedicated to image visualization. It is therefore one of the axes of development for the near future, to create specific *viewers* associated with *Vobi One processes* and image datatypes, and that fully exploit the power of Anatomist while facilitating its use for the end users.

Second, we are also working on new methodological developments to improve the efficiency of our linear model for various types of data. Indeed, this framework has been successfully applied on anesthetized rat (unpublished), cat (unpublished), and awake monkey (Reynaud et al., 2011, 2012). From our experience, the key point to successfully use this framework in various species is to properly estimate the noise components that are specific to each preparation, being physiological, environmental or instrumental. For instance, when using intrinsic optical imaging in rats, we have observed that the variations in heartbeat and respiration frequencies from trial to trial prevent from building a model that would be valid for all trials of a given session. We are therefore developing methods to estimate the heartbeat and respiration frequencies from physiological recordings at the level of the individual trial. These parameters are then used to build a model adapted to each trial. We believe that this new functionality will greatly facilitate the construction of accurate linear models over other animal models.

### 4.3. Distribution and collaborative development

*Vobi One* is available through our website^5^. This site is also our internal software development plateform, which uses a Trac^6^ system based on Mercurial^7^ for version control. The software package is distributed as a tarball that just needs to be unpacked in the BrainVISA installation directory. It comes with detailed installation instructions and a user's guide that takes the reader step by step into the BrainVISA environment and each of the *Vobi One* processes. A full dataset is also provided, which is one session of the data that served to benchmark our linear model in (Reynaud et al., 2011). We strongly hope that researchers will benefit from using *Vobi One* and that developers will be interested in further contributing to its development. Short term objectives for such collaborative development would obviously be to support additional data formats to better answer the needs of the community, and also to add other data processing techniques such as those based on source separation algorithms for instance GIF (Yokoo, 2001), or TSCA (Blumenfeld, 2010; Omer et al., 2013). Objectives in the longer term could be to provide data processing methods for other types of optical imaging data as we discussed above. We will be available to help both researchers and developers in order to further improve the *Vobi One* toolbox in any of these directions.

## 5. Conclusion

We have described *Vobi One*, the first open source software suite dedicated to the analysis of functional optical brain imaging operating at the mesoscopic scale. *Vobi One* is a toolbox of the BrainVISA neuroimaging software environment. As such, its developers can directly make use of BrainVISA's image processing library and its API, while its users benefit from its graphical user interface, its built-in database support and its interoperability capability. Advanced users can also write Python scripts to fully operate the toolbox, thus facilitating the production of reproducible research. Two main types of analysis methods are available in *Vobi One*: (1) the standard method based on divisive normalization to baseline level followed by subtraction of blank trials; and (2) a framework based on a thorough modeling of all noise sources and a multiple linear regression, as described in Reynaud et al. (2011). Although still in a fairly early phase of development, *Vobi One* now offers a solid, yet limited, set of features, and we think that it can therefore be useful to the small research community that uses functional optical imaging. We also hope that by making it open source, other developers will contribute to strengthen its content, by for instance providing the possibility to use blind source separation methods.

### Conflict of interest statement

The authors declare that the research was conducted in the absence of any commercial or financial relationships that could be construed as a potential conflict of interest.

## Footnotes

^1^http://www.brainvisa.info.

^2^http://brainvisa.info/soma/soma-workflow.

^3^http://www.python.org/.

^4^http://www.numpy.org/.

^5^https://trac.int.univ-amu.fr/vobi_one.

^6^http://trac.edgewall.org/.

^7^http://mercurial.selenic.com/.
